# Effects of Climate Change on the Immune System: A Narrative Review

**DOI:** 10.1002/hsr2.70627

**Published:** 2025-04-18

**Authors:** Luisa Imberti, Giorgio Tiecco, Jacopo Logiudice, Francesco Castelli, Eugenia Quiros‐Roldan

**Affiliations:** ^1^ Section of Microbiology University of Brescia Brescia Italy; ^2^ Department of Clinical and Experimental Sciences, Unit of Infectious and Tropical Diseases University of Brescia and ASST Spedali Civili di Brescia Brescia Italy

**Keywords:** climate, climate change, immune system, immunity

## Abstract

**Background and Aims:**

Human activities have greatly influenced global temperatures, leading to climate change and global warming. This narrative review aims to explore the relationship between climate change and the immune system, focusing on how environmental stressors can affect immune regulation, leading to both hyperactivity and suppression.

**Methods:**

A comprehensive search was conducted in PubMed and Google Scholar for peer‐reviewed studies published up to June 2024. The search terms included “climate change,” “human health,” “infection,” “immunity,” and “disease.” Inclusion criteria were based on relevance, originality, and accessibility.

**Results:**

Exposure to elevated temperatures can significantly impair immune system cells, leading to an overproduction of signaling molecules that promote inflammation. Temperature fluctuations have been shown to influence various aspects of the adaptive immune response, including immune cell mobilization, antigen processing and presentation, lymphocyte trafficking and activation, and the functionality of B and T cells. Notably, some research suggests that heat stress negatively impacts B lymphocyte differentiation, replication, and proportion, resulting in decreased immunoglobulin and cytokine production, and contributing to immunosuppression. Additionally, climate change‐related exposures can compromise epithelial barriers in the skin, lungs, and gut, leading to microbial dysbiosis, and immune dysregulation. Furthermore, environmental factors such as temperature variations, humidity, and air pollutant levels may exacerbate the prevalence of infectious diseases, including measles and HIV, with varying impacts on acute, chronic, and latent infections, further contributing to immune variability.

**Conclusion:**

Climate change, particularly increased temperatures, significantly impacts immune system function, leading to both heightened inflammatory responses, and immunosuppression. Future research should focus on developing comprehensive and sustainable management strategies to enhance health resilience in the face of ongoing climatic changes.

## 1 Background

Human activities have strongly impacted global temperatures, resulting in global warming and climatic changes [1]. According to the World Meteorological Organization (WMO), climate change “is the term used to describe changes in the state of the climate that can be identified by changes in the average and/or the variability of its properties and that persists for an extended period, typically decades or longer” [2]. This alteration in weather patterns can increase the severity and frequency of heatwaves, often accompanied by high humidity [3]. These changes contribute to environmental disaster and stress on living organisms. Heatwaves are becoming more frequent and intense globally, with an increasing number of warm days in most regions. Additionally, extreme climate events such as droughts, heat waves, dust storms, floods, typhoons, or hurricanes are changing in frequency and geographic distribution [4]. According to the United Nations Intergovernmental Panel on Climate Change (IPCC), without adequate climate policies, global warming projections indicate a temperature increase of between 3.1°C and 5.1°C by 2100 compared to preindustrial levels [5]. The effects of climate change disproportionately impact low‐income communities and countries, which are often the least responsible for contributing to its causes and have weaker health systems [6]. Vulnerable populations, including the elderly, children, and individuals with chronic diseases, are particularly affected [7, 8].

Climate change and related extreme weather events represent one of the most pressing global challenges of the 21st century due to their complex impact on humans. At the 54th Annual Meeting of the World Economic Forum in January 2024, the impact of climate change on nature, global economy, and human health and healthcare systems was widely discussed. Climate change is estimated to strain global health systems by 2050, potentially causing an additional 14.5 million deaths and imposing a burden of $10.1 trillion due to climate‐induced impacts, underscoring the need for healthcare system adaptation [9].

Climatic change contributes to an array of direct and indirect health effects including malnutrition, mental health challenges, cancer, and infectious diseases with involvement of many organs [10]. Extreme weather events, which also increase health risks, can indirectly affect health determinants such as clean water, nutritious foods, and access to healthcare. For example, the impact of climate change on vector‐borne diseases is rapidly increasing, with an additional 500 million people potentially at risk of exposure by 2050 [11, 12, 13, 14, 15]. Moreover, shifts in weather patterns are also driving substantial changes in the global distribution of pathogens, hosts and diseases reservoirs [14, 15].

**Figure 1:**
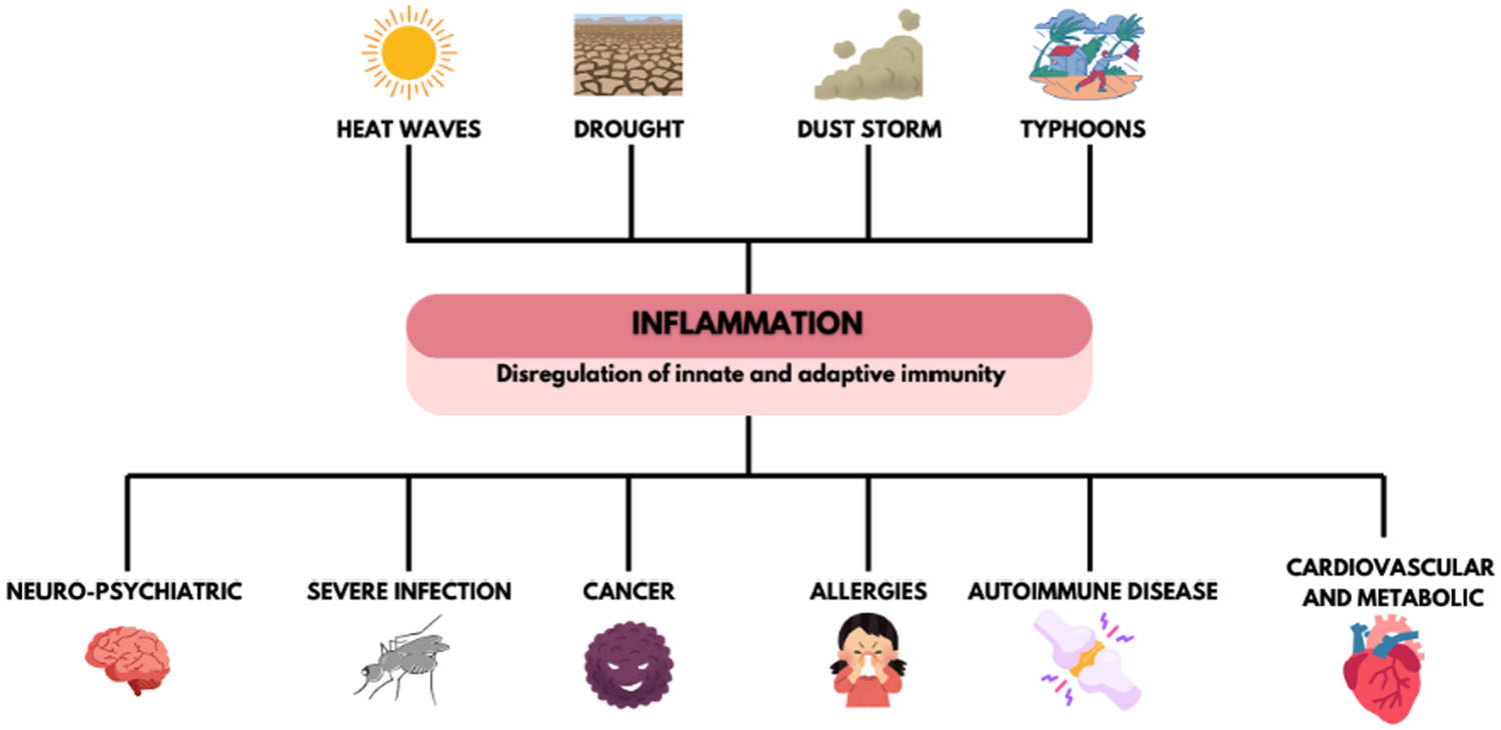
Climate events, immune dysregulation, and their effects [16].

Therefore, climate change is a multifaceted global challenge with far‐reaching consequences for human health and well‐being [14]. The health impacts of climate change are pervasive and multisystemic, affecting most, if not all, organ systems. Cerebrovascular, cardiovascular, metabolic, renal, infectious respiratory diseases as well as malignancy and autoimmunity are expected to increase due to climate change [16]. (Figure 1).

Several biological mechanisms may explain the heat‐related health risks, particularly for the elderly. Patients with neuropsychiatric diseases are especially vulnerable, as their ability to adapt and cope with climate change is already reduced. The psychological effects of climate change—whether from accepting its inevitability or experiencing its consequences—can further heighten the risk of brain diseases, similar to what is observed in post‐traumatic stress disorder [17]. When body temperature rises, blood flow shifts from vital organs to the skin to promote cooling. However, excessive blood diversion can impair thermoregulation, placing added stress on the heart and lungs. Other factors, such as increased blood viscosity, elevated cholesterol levels in warmer conditions, and a higher sweating threshold, may also contribute to these risks [18].

Climate change may also increase cancer risk due to greater ultraviolet exposure, higher levels of air pollution and toxic chemicals, and reduced access to cancer screening and treatment. Additionally, temperature shifts affect the timing and duration of pollen seasons, leading to increased pollen production and potential airway obstruction, which can trigger asthma attacks [19].

One significant aspect of climate change's impact is its influence on the immune system. The pathological conditions mentioned can occur or exacerbate due to both the overactivity (upregulation) and suppression (downregulation) of the immune system [20]. This study aims to investigate aspects of the intricate relationship between climatic change and the immune system.

## 2 Temperature and Immune System

Environmental factors such as temperature, humidity, air pollutant concentrations, ocean pH, and salinity can stress both animals and humans, triggering complex pathways the mediate the immune response [21]. The consequences of increased temperatures of body are the most extensively studied. Thermoregulation is essential for maintaining the core body temperature around 37°C, supporting cellular functions. Although the body has evolved mechanisms to adapt to environmental temperature fluctuations, the increasing frequency and intensity of heatwaves due to global warming have significant short‐ and long‐term effects on human health.

In humans, an observational, longitudinal, cohort study of over 35,000 outpatients at a large academic hospital found that a 1°C increase in body temperature correlated with a 3.5% higher mortality after 1 year, even when comparing for age, sex, disparity, and time of day which are known factors influencing body temperature [22]. Furthermore, a trend towards an increase in the prevalence of diseases related to immune dysregulation with increasing temperature was observed [16, 23].

However, while the effects of heat on cardiovascular, respiratory, endocrine, urinary and reproductive systems have been extensively studied in humans [24], The effects of temperature changes on the human immune system are less studied. Research has primarily focused on immune system changes during fever and in vitro experiments, with most available information coming from animal studies [25]. For instance, increased environmental temperatures raise the body temperature of insects, altering some physiological processes such as immune response capacity and accelerating immune senescence, thereby decoupling physiology from chronological age [26]. In mice, environmental temperature also influences immune responses because animals housed at the thermoneutral temperature of 31°C respond to bacterial lipopolysaccharides challenge with hyperthermic fever, whereas the same immune challenge at a temperature of 26°C results in transient hypothermia [27].

In humans there is indirect evidence of dysregulation of the immune system during heat waves. A strong association between heat waves and emergency department visits for intestinal infections was described [28], also a high incidence of urinary, blood or lung infections have been reported in patients hospitalized for heat stroke during sustained heatwaves [29, 30, 31]. Data about the immune response to heat in humans comes from studies focused on the effects of short‐term heat exposure, such as during fever, on the immune system. The heat shock response (HSR) exemplifies the impact of temperature on immunity, a phenomenon observed in virtually all living organisms [32].

## 3 Increased Temperature and Innate Immunity

There is evidence that exposure of the human body to high temperatures may impair the cells of the immune system and over‐produce signaling molecules that lead to inflammation. Presbitero et al investigated the relationship between core temperature and the human innate immune response during physical activities demonstrating that the immune response is influenced differently by varying levels of heat exposure [24]. Their study found that moderate thermal stress, which elevates body temperature to 37°C–38°C, improves the efficiency of the innate immune response. Conversely, when core temperature exceeds 38°C, it has detrimental effects. One explanation for this phenomenon is that low temperature nudges the innate immune system into activation and improves the efficiency of its response. Conversely, if the body has already been exposed to inflammation‐triggering agents and has activated its immune response to resolve the inflammation, a significant increase in temperature can intensify this process. Instead of aiding resolution, the added heat can further stimulate inflammation, potentially preventing the body from effectively resolving the ongoing immune response [24].

Similarly, preliminary research involving 624 adults indicated that every 5°C increase in Universal Thermal Climate Index, a bioclimatic index for describing physiological comfort under specific meteorological condition, resulted in elevated blood levels of key markers of innate immune system, such as monocytes (4.2%), eosinophils (9.5%), natural killer T cells (9.9%), and tumor necrosis factor (TNF‐α) (7%) [33]. These changes reflect an activation of the innate immune system, inducing a rapid, nonspecific inflammatory response that protects the body from pathogens and injuries.

**Figure 2:**
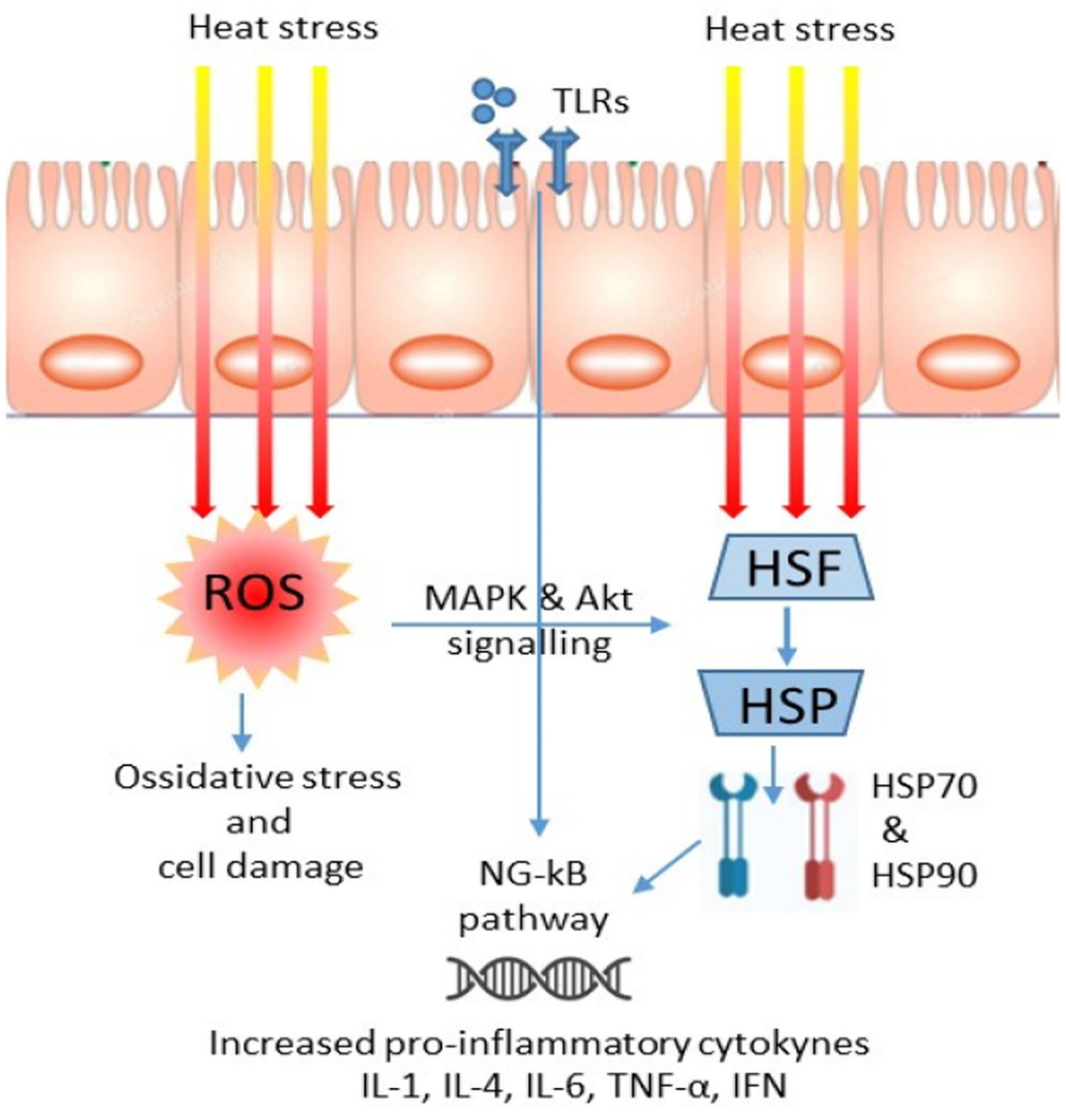
Heat stress increases the production of ROS activating the heat shock response pathway, resulting in the release of proinflammatory cytokines.

The cellular response to thermal stress in mammalian organisms is controlled and mediated by heat shock transcription factors (HSF) [34]. Activation of HSFs enhances the transcription of heat shock proteins (HSPs) mRNA. In vitro studies have shown that heat shock activates reactive oxygen species (ROS), which stimulate p38 mitogen‐activated protein kinases (MAPK) and serine/threonine kinase (Akt) signaling, leading to HSF and HSP activation [35]. HSPs, particularly HSP70 and HSP90, play crucial roles in protein metabolism, apoptosis, inflammation, and natural immune functions by inducing the release of pro‐inflammatory cytokines such as interleukin (IL)‐1, IL‐4, IL‐6, and TNF‐α (Figure 2) [36].

Heat stress also induces innate immunity and inflammatory reactions through the upregulation of the toll‐like receptor 4 (TLR4) signaling pathway. This stress‐related biosensor, acting on NF‐κB, the main nuclear transcription factor of the natural immunity and inflammatory response, changes the expression of a series of inflammatory related factors such as IL‐1β, IL‐6, TNF‐α, and type 1 interferons (IFN; Figure 2) [37]. The immune response to stress varies depending on the degree of stress and cell type. All leukocyte subtypes react to body temperatures up to 39°C by inducing HSP70 expression [38]. Heat stress can reduce the absolute number of white blood cells and lymphocytes but increase neutrophils, monocytes, and the neutrophil‐to‐lymphocyte ratio. This ratio is used to measure heat stress effects on the immune system, predict immune disease risk, and detect infectious and inflammatory diseases early [39].

During febrile episodes, the temperature of the whole body increases with a granulocyte‐colony‐stimulating factor (G‐CSF)‐induced proliferation of hematopoietic stem cells and neutrophil progenitors in the bone marrow. Therefore, it substantially increases the number of neutrophils in the blood and their recruitment to infection sites in a CXC‐chemokine ligand 8 (CXCL8)‐dependent manner [32]. Furthermore, the elevated respiratory burst induced by thermal stress, increases the bacteriolytic activity of neutrophil in the site of infection.

Heat also increases the expression of MHC class I and class II molecules and CD80 e CD86 costimulatory molecules, as well as CC‐chemokine receptor 7 (CCR7)‐dependent migration in mature dendritic cells, that serve as conduit to draining lymph nodes. It also enhances natural killer (NK) cell cytolytic activity by inducing MHC class I polypeptide‐related sequence A expression on target cells, such as cancer cells, and increases the phagocytic potential of macrophages and dendritic cells. Additionally, heat‐exposed dendritic cells are more efficient at cross‐presenting antigens and inducing T helper 1 (Th1) cell polarization [40].

The hypothalamic‐pituitary‐adrenal and sympathetic‐adrenal‐medullary axes are activated during heat stress to maintain homeostasis, producing cortisol, which has different effects in acute versus chronic phases. For instance, in animals, acute stress‐induced cortisol stimulates the immune system, whereas chronic stress‐induced cortisol suppresses it by inhibiting pro‐inflammatory cytokines such as IL‐4, IL‐5, IL‐6, IL‐12, IFN‐γ, and TNF‐α, resulting in an increased susceptibility to disease and immune challenges [41]. Additionally, also anti‐inflammatory cytokines like IL‐10, IL‐1 receptor antagonist (IL‐1RA), and soluble TNF receptors increase during acute heat stress in both animal models and human [42]. In threatening situations, acute stress may enhance survival. However, when stress responses are activated chronically or inappropriately, they can disrupt normal bodily functions and increase the risk of disease, even in ways unrelated to the initial threat.

## 4 Increased Temperature and Adaptive Immunity

The adaptive immune response has evolved over hundreds of millions of years to eliminate invading pathogens and their toxic products, as well as a multitude of discrete antigens present in the environment, including those in food and air. Changes in temperature can influence various aspects of the adaptive immune response, affecting immune cell mobilization, antigen processing, and presentation, lymphocyte trafficking and activation, and the functionality of B and T cells [43].

During infection, elevated body temperatures enhance lymphocyte trafficking across high endothelial venules (HEVs) of peripheral lymph nodes through increased expression of α4 integrin and HSP90, which promote T‐lymphocyte trafficking during infection [44], and by interfering on several steps of the lymphocyte adhesion cascade [32]. For instance, it has been demonstrated that heat treatment of lymphocytes increases the frequency of l‐selectin‐dependent tethering and rolling interactions. Febrile‐range temperatures enhance the stable arrest of lymphocytes on HEVs by increasing the intravascular density of CC‐chemokine ligand 21 (CCL21) on the heparan sulfate‐rich glycocalyx, and intercellular adhesion molecule 1 (ICAM1), which also supports lymphocyte crawling to inter‐endothelial cell junctions and trans‐endothelial migration.

Increased trafficking of immune cells to inflamed tissues is an important step in effective immune surveillance during febrile inflammatory responses to infections. Once lymphocytes manage to enter into lymphoid organs there is evidence that their ability to respond to stimulatory signals is also enhanced by febrile temperatures.

For instance, within lymphoid organs, heat directly affects T cells by pre‐clustering immunological synapse (T ‐cell receptor β‐chain and CD8, for instance), which may be a mechanism by which T cells can achieve a high sensitivity [40]. T cells are capable of respond prominently to temperature changes because heat shock factor 1 (HSF1) is induced at a lower temperature in T cells (39°C) than in B cells (42°C) [45]. In particular, in vitro studies show rapid and significant proliferation of T cells at increased body temperatures [46]. Exposure to 39°C, although having limited effects on proliferation or activation marker expression, augments the metabolic activity and effector functions of activated CD8 T cells [47]. In contrast, naïve CD4 T cells undergo transcriptional reprogramming, favoring a Th2 phenotype over Th1 cells producing IFNγ [48]. Heat also induces changes in CD4 cell plasma membrane fluidity and macromolecular clustering, reducing the requirement for CD28 stimulation for IL‐2 production [40]. Elevated temperature also affects Th17 cell differentiation, increasing IL17a production, and enhancing neutrophil invasion into bronchoalveolar lavage fluid in a mouse model of allergic airway inflammation [49]. A potential explanation is that high temperatures during CD8 + T cell activation modify mitochondrial activity and cause extracellular acidification, a pathway particularly enhanced in Th17 cells [43].

The relationship between heat stress and B lymphocytes remains controversial. Ex vivo heat treatment of B cells has been shown to nearly double their ability to bind to HEVs in vitro and enhance their migration to lymph nodes and Peyer's patches in vivo. Similar to T cells, blood‐borne B cells enter these sites through a well‐defined adhesion cascade. Additionally, long‐term heat stress has been found to increase the proportion of B lymphocytes in the spleen of pigs, suggesting that heat stress may enhance adaptive immunity in the spleen [50]. However, other studies indicate that heat stress reduces B lymphocyte differentiation, replication, and proportion in animals, leading to lower immunoglobulin and cytokine levels, and causing immunosuppression [51]. In addition, a recent research shows a 6.8% decrease in B‐cell production due to heat stress [33], and lower IgM levels, though not IgG or IgA, in foundry workers [39].

The complexity of the pathways activated during the high temperature response is mirrored by the multitude of molecules and cell types involved and many of the mechanisms behind these associations remain to be understood whether they are activated also as a consequence of the increase of environmental temperature.

It is important to fully understand at the cellular level how heat affects the inner workings of the human immune system response because heat waves represent a challenge for public health and should be specifically addressed by future research and public health policies.

## 5 Climate Change and Barriers Disruption

Innate immunity also includes physical barriers consisting of epithelial and mucosal (respiratory and gut) surfaces, along with mucus and resident immune cells. Additionally, although not included as a component of the innate immunity, commensal microorganisms, or microbiota, form an essential biological barrier [52]. The interplay between the microbiota and immune system is a key factor for health, and imbalances in microbiota‐immunity interactions under certain environmental conditions are implicated in numerous immune‐mediated disorders [53]. Epithelial barriers of the skin, lung, and gut serve as the first line of human defense. Climate variables including temperature, humidity, ultraviolet radiation, and air pollution may modulate microbiome and be relevant for human health [54].

**Figure 3:**
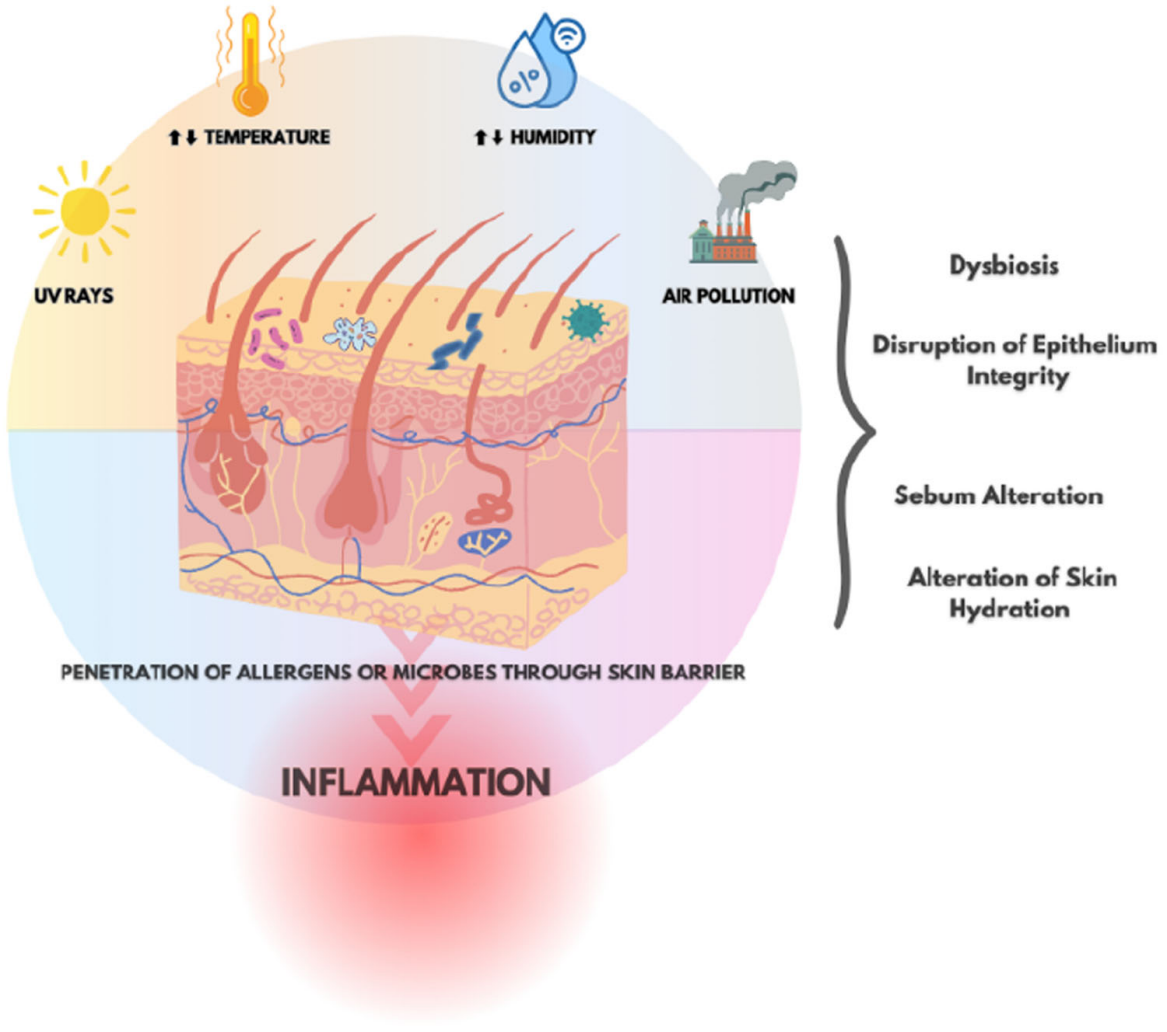
External environment affected by climate change.

The skin, the body's primary interface with the external environment, is particularly affected by climate change. Environmental changes, such as variations in temperature, humidity, and air pollutant concentrations can alter epithelial composition, compromise epithelial integrity, induce microbial dysbiosis and immune dysregulation, thus contributing to the emergence of allergic and autoimmune conditions and alter the course of skin diseases (Figure 3) [55].

Epithelial damage facilitates the penetration of allergens and microbes triggering proinflammatory responses. Heat waves impact skin health due to the fundamental role of the epithelial barrier in thermoregulation through vaso‐reaction and sweat secretion. Populations with impaired thermoregulation, such as infants, elderly individuals, and patients with chronic cardiovascular or kidney diseases, are especially vulnerable to heat waves [56, 57]. Higher ambient temperatures exacerbate conditions such as intertrigo, especially in individuals with obesity and diabetes, as well as contact dermatitis [58]. A hot environment triggers flares of rosacea, cholinergic urticaria, and heat urticaria. Hyperhidrosis, a common consequence of high temperatures, leads to acantholytic dermatosis (Grover disease) [59]. Additionally, high humidity and sweating can provoke dermatophyte infections and folliculitis [60].

Environmental humidity can also affect skin hydration. Low humidity levels result in dryness, increased permeability to allergens and antigens, and the production of inflammatory mediators [61, 62, 63, 64]. This issue is particularly pronounced in older adults, who are more sensitive to changes in skin and mucous membrane humidity and have diminished perception [65].

Increased levels of air pollution, including particulate matter (PM), can induce oxidative stress and inflammation in the skin [66] heightening the risk of allergic skin diseases through immune dysregulation and barrier dysfunction. PM may cause skin barrier dysfunction by directly producing ROS or inducing ROS generation through intracellular mitochondrial damage and autophagy. This can activate downstream MAPK signaling pathways, the production transcription factors (NF‐κB and AP‐1, for instance), which eventually leads to upregulation of cytokines such as TNF‐α, IL‐1α, IL‐6, that induce inflammatory skin diseases.

Air pollution can also alter the skin microbiome, increasing allergic susceptibility [61]. Interestingly, prenatal air pollutant exposure can modify children's immune responses, predisposing them to atopic dermatitis postnatally [67]. Recently it has been suggested that prenatal and postnatal pollutant exposure is linked to accelerated morbidity and mortality in various health conditions [68].

Ultraviolet (UV) radiation, largely independent of temperature and humidity, can also impact skin health. Heat waves may increase UV exposure as individuals wear less clothing and spend longer periods outdoors. UV radiation can cause mutations leading to skin cancers and induce immunosuppression by altering the number, phenotype, and function of circulating neutrophils, T cells, B cells, and NK cells [69].

Mucosal respiratory surfaces can be altered by ozone (O_3_), a pollutant that increases with climate warming. O_3_ induces oxidative damage through ROS and disrupts the immune system by altering regulatory T cells, exacerbating pre‐existing respiratory diseases [70].

The gut barrier, another functional defensive unit, comprises a physical barrier of tightly connected intestinal epithelial cells and immune cells that manage the immune tolerance and the immune responses to pathogens, along with a biological barrier of gut microbiota. Environmental heat directs blood flow to the body surface for radiant heat loss, causing vasoconstriction in the gut mucosa and increased intestinal permeability [71]. Elevated plasma endotoxin levels, an indirect measure of intestinal permeability, have been observed in individuals exposed to high temperatures [72, 73]. The gastrointestinal tract's dense microbial community influences host metabolism, immunity, and neuro‐behavior. However, gut microbiota can affect and be affected by extreme temperatures [74, 75].

There are evidence that climate change and air pollution can also alter environmental microbiota, impacting infant colonization and subsequent microbiota in the skin, respiratory system, and gut [68]. This dysbiosis can lead to significant changes in the infant immune system, including altered TLR expression, T‐cell function, antiviral responses, and allergic sensitization. Loss of biodiversity and limited microbial exposure are major factors impairing tolerogenic immune development [68].

While air pollutants are traditionally linked to respiratory and cardiovascular issues, recent studies indicate potential correlations with gastrointestinal disorders. Long‐term exposure to ambient air pollutants is associated with an increased risk of peptic ulcers and chronic gastritis [76]. Ingested PM alters gut microbiome composition, increasing gut permeability and decreasing colonic motility in mice, with notable changes in the relative abundances of Bacteroidetes, Firmicutes, and Verrucomicrobia [77].

Most of microbiome studies mentioned above focused on single anatomic sites although the gut‐lung‐skin axis is emerging as field for exploration. As example it has been described as gut microbiota can translocate across a perturbed intestinal barrier and through mesenteric vessels disturb skin or lung microbiome and impact in immunity [78, 79] Dysbiosis of this homeostasis is key to understanding the development of disease.

## 6 Climate Change and Immune Components Variability

**Figure 4:**
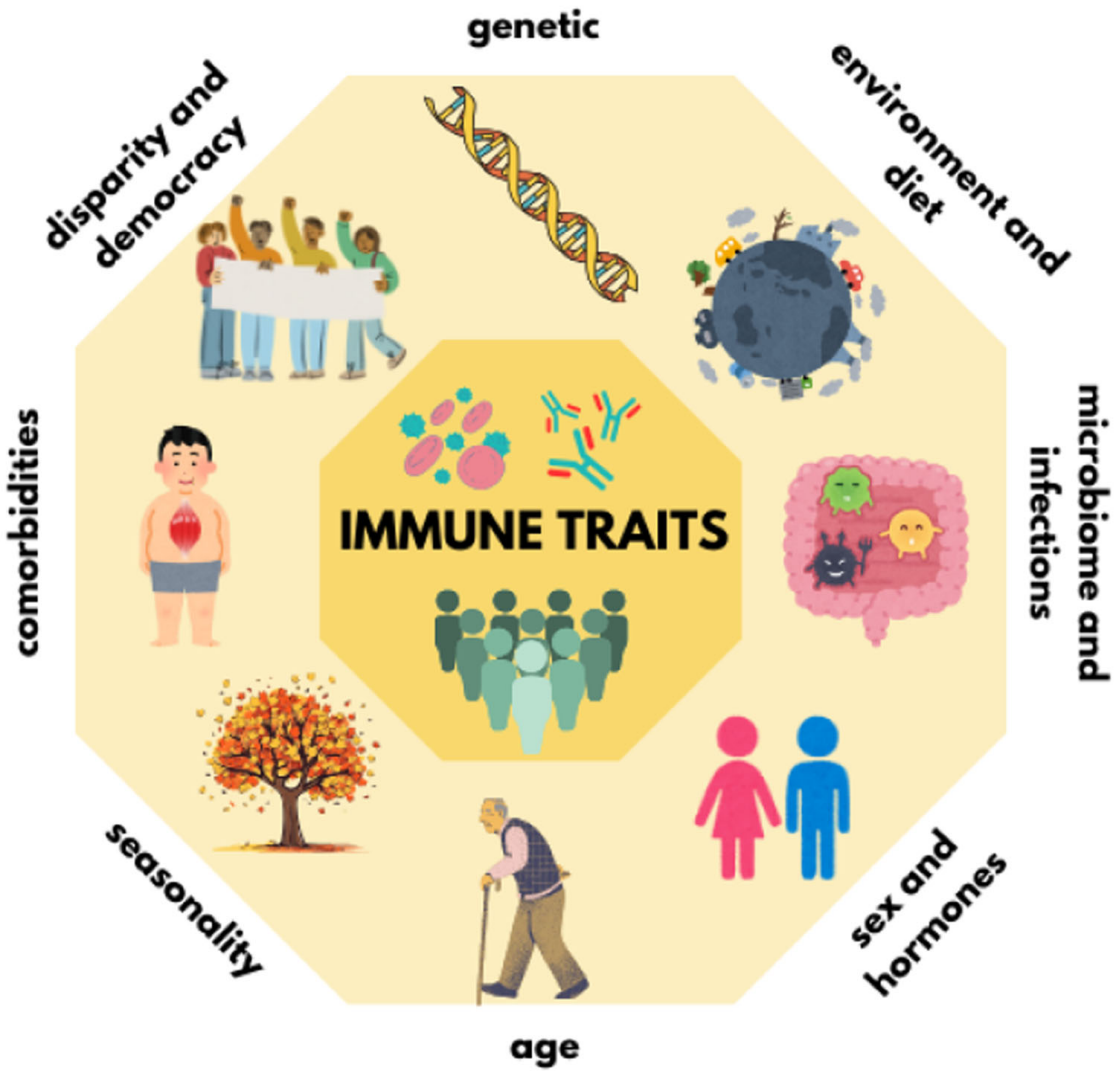
Intrinsic and extrinsic factors affecting immune variability.

The human immune system, while relatively stable within an individual, exhibits highly variability among individuals. This variability influences differential risks for infection, autoimmunity development, and for therapeutic response. Immune heterogeneity arises from both heritable and non‐heritable (Figure 4), as well as intrinsic and extrinsic, factors, with non‐heritable influences, such as environmental exposures, accounting for most variations [80]. Knowledge of the factors that influence the heterogeneity of the immune system in different individuals could help to understand how climate change may influence the reactivity of the immune system. This has important implications for immune competence and individual response to injuries. As illustrated in Figure 3, several conditions induce the immune variability: (1) genetic variables, such as ancestry or predisposition to chronic disease and/or comorbidities, such as type I diabetes, are associated to immune heterogeneity; (2) health‐impacting behaviors, such as microbial exposures as diet, which reflect on gut microbiota composition, as well as vaccination and smoking; (3) biological mediators, such as sex and age; (4) chronic disease and/or comorbidities which can be driven by external factors, such as obesity; (5) nonconstitutional, extrinsic environmental factors, such as socioeconomic status, which can impact access to healthy foods and medical care; and (6) the effect of democracy on population health.

Climate changes can be included among specific factors that may increase or decrease susceptibility to infections within a population, thus influencing infection resistance or disease tolerance. For instance, between the three and six billion people that are living in regions highly susceptible to climate change, many lack common vaccinations [81]. Climate change can increase the prevalence of infectious diseases, such as measles [82] and HIV [81] with acute, chronic, and latent infections that differentially impact immune variability. It has been proposed that this different impact on immune system variability may be related to the age of the infected subject. Therefore, the measles virus that, in the absence of vaccination, primarily encountered in childhood can have a major influence on broad immunity, and indeed, acute measles infection in unvaccinated children can eliminate 11%–73% of the pre‐existing antibody response [83]. On the contrary, chronic HIV infection impacts immune cell repertoires, HCV has an indirect effects, such as a bystander effect, on the naïve T cell repertoire, and latent cytomegalovirus infection modulates NK and T‐cell populations and may increase the risk of progression to tuberculosis and may compromise the immune response to vaccines [84].

**Figure 5:**
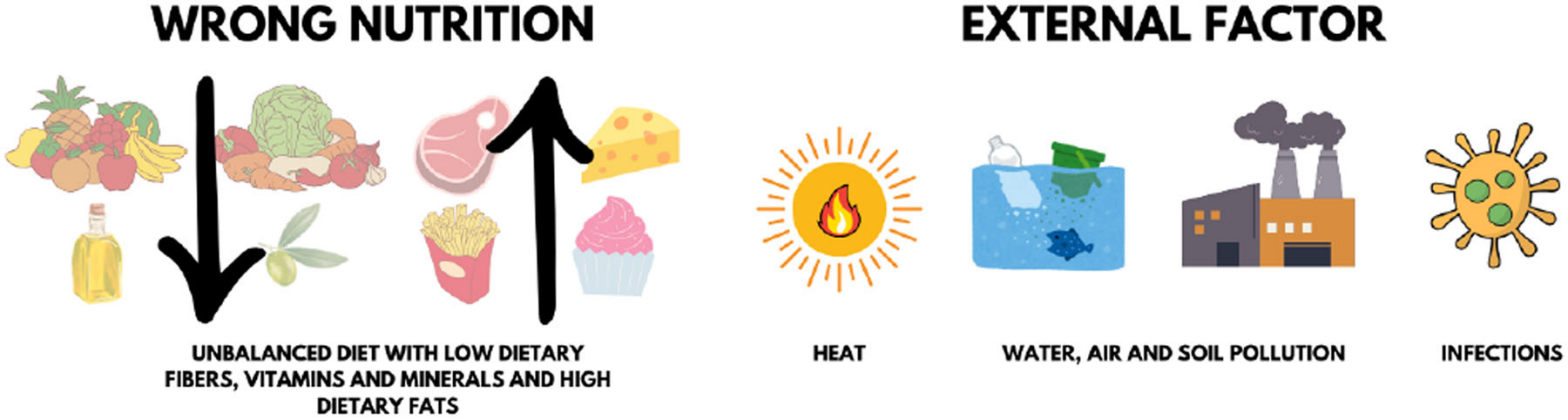
Dietary and external factors influencing immune system composition and functionality.

Proper nutrition, especially a well‐balanced and nutrient‐rich diet, is essential for overall health and immune function, highlighting the connection between food and the immune system [85]. Nutritional components, such as antioxidants and anti‐inflammatory compounds found in fruits, vegetables, and other plant‐based foods, as well as an adequate intake of essential nutrients, including vitamins (such as vitamins C, D, and E), minerals (such as zinc and selenium) and other bioactive compounds, are necessary to maintain a good immune function (Figure 5) [85].

However, extreme climate events affecting terrestrial and marine food supplies and reduced nutrient concentrations in crops due to high CO_2_ levels can lead to malnutrition, impacting immune competence. Changing of food crop quality may also alter the composition of intestinal microbiota, that is known to have a strong interaction with the immune system. Malnutrition, alongside low socioeconomic status‐linked factors, such as pollution, exposure to stress and smoking, is associated with chronic inflammation and immune system deterioration, contributing to increased disease risks, including Cryptosporidium, measles, tuberculosis, and cholera infections in food‐poor populations [14, 86, 87].

Temperature rhythms can vary depending on age and sex. Body temperatures decrease with age with a difference of 0.3°C between individuals of 20–59 vs > 60 years, after controlling for sex, body mass index, and white blood cell count. This is consistent with low body temperature as a biomarker for longevity. However, females are + 0.1–0.5°C warmer than males on average [25]. This has been attributed to hormone levels since females have monthly circadian temperature cycles, and temperature changes that coincide with the start and cessation of menses. They also have a peak of + 0.6°C at ovulation, when aspects of the innate, humoral, and cell‐mediated immune system are suppressed in the reproductive tract by sex hormones to optimize conditions for procreation [88]. Hormonal contraception can maintain elevated hormone levels, increasing infection risks and susceptibility to autoimmune diseases such as Crohn's disease, systemic lupus erythematosus, and multiple sclerosis [89].

Seasonal variations also influence the immune system, with respiratory virus infections more common in winter and polio in summer. A recent study performed in 329,261 participants demonstrated that neutrophil and lymphocyte counts were higher in winter and lower in autumn, while monocyte counts and antibody titers showed no significant seasonal pattern [90]. These variations suggest that humans have some capacity for regulation of immune cells that could contribute to seasonality and daily susceptibility to infections.

Climate change impacts resource availability and economic stability, exacerbating inequality and poverty, potentially leading to social unrest, political instability, conflict, migration, and threats to democracy. Democracies, particularly high‐income ones, generally have better health outcomes due to the provision of health‐promoting resources and services, which can influence immune system heterogeneity through health programs and vaccination campaigns [91].

As described above, climate can have a great impact on human health with the immune system directly and indirectly involved. In this panorama it is particularly alarming the significant changes in the pattern of infectious disease occurrence in various localities around the globe [15]. Pathogens and vectors that carry them are migrating to new habitats and some are strengthened under adverse weather conditions. For example, the risk of West Nile virus outbreaks in Europe is projected to increase fivefold by 2040–2060 compared to 2000–2020 [92]. After 50 years that Italy was officially declared malaria free by the World Health Organization (WHO), *Anopheles sacharovi*, the vector of malaria, has reappeared in Italy [93]. *Candida auris* has developed the ability to adapt and survive at high temperatures (above 37°C) [94]. Increased antimicrobial drug use in humans and animals as a consequence of climate change point towards an increasing antimicrobial resistance burden [95]. Climate change increases the risk or exacerbation of 58% of infectious diseases [14].

Understanding the immunological effects of climate change is crucial for developing and strengthening effective management strategies, ensuring health and welfare amidst climate challenges, and adopting a One Health approach to climate change adaptation, which encompasses human health, environmental health, and agricultural ecosystems.

To better understand and predict the global impact of climate change on immunity, more comprehensive, collaborative, and standardized studies are needed. Research should not be limited to low‐disease burden countries with high‐quality healthcare access but must also address neglected diseases and vulnerable populations. It is crucial to assess personal and social risks, identify working conditions that increase susceptibility to heat‐related illnesses, and provide clear guidance for at‐risk groups. Additionally, studies should evaluate the effectiveness of preventive measures to mitigate these health risks.

## 7 Search Strategy, Selection Criteria, Strengths and Limitations

We conducted a search on PubMed and Google Scholar for peer‐reviewed, English‐language quantitative studies published up to June 1, 2024, that investigated the impact of climate change on human health. We identified relevant references from MEDLINE and PubMed using the search terms: “climate change” AND “human health” OR “infection” OR “immunity” OR “disease.” We included studies by first screening titles and abstracts, followed by a full‐text review of the selected studies. This process was independently performed by two authors, with any disagreements resolved through discussion. This study does not constitute a systematic review; rather, the final selection of references was curated based on criteria including the publication date, originality, accessibility, and relevance to the scope of this narrative review. The corresponding author applied the SANRA (Scale for the Assessment of Narrative Review Articles) six‐items scales [83] to enhance rigor and reliability in our work. The main strengths of narrative reviews are their flexibility and practicality, offering readable syntheses, interpretations, and critiques of literature on a topic. However, they are limited by potential author bias, lack of evidence‐based synthesis for focused questions, though they can still provide meaningful and insightful summaries.

## Author Contributions

**Luisa Imberti:** conceptualization, data curation, methodology, supervision, validation, visualization, writing – review and editing, writing – original draft, formal analysis. **Giorgio Tiecco:** validation. visualization. writing – review and editing. writingm – original draft. jacopo logiudice: validation, visualization, writing – review and editing. **Francesco Castelli:** validation, visualization, writing – review and editing. **Eugenia Quiros‐Roldan:** conceptualization, data curation, funding acquisition, methodology, supervision, validation, writing – review and editing, visualization, writing – original draft, formal analysis.

## Disclosure

During the preparation of the manuscript, the authors used ChatGPT, provided by OpenAI, to improve grammar and readability of the paper. After using this service, the authors reviewed and edited the content as needed. The authors take full responsibility for the content of the publication.

## Conflicts of Interest

The authors declare no conflicts of interest.

## Transparency Statement

The lead author Eugenia Quiros‐Roldan affirms that this manuscript is an honest, accurate, and transparent account of the study being reported; that no important aspects of the study have been omitted; and that any discrepancies from the study as planned (and, if relevant, registered) have been explained.

## Data Availability

The authors have nothing to report.
